# Assessment of Bleached and Unbleached Nanofibers from Pistachio Shells for Nanopaper Making

**DOI:** 10.3390/molecules26051371

**Published:** 2021-03-04

**Authors:** Eduardo Robles, Nagore Izaguirre, Ander Martin, Dimitra Moschou, Jalel Labidi

**Affiliations:** 1 Biorefinery Processes Research Group, Chemical & Environmental Engineering Department, Faculty of Engineering, Gipuzkoa, University of the Basque Country UPV/EHU, Plaza Europa 1, 20018 Donostia, Spain; nagore.izaguirre@ehu.eus (N.I.); amartin261@ikasle.ehu.eus (A.M.); dimitramosx21@gmail.com (D.M.); jalel.labidi@ehu.eus (J.L.); 2 University of Pau and the Adour Region, E2S UPPA, CNRS, Institute of Analytical and Physicochemi-cal Sciences for the Environment and Materials (IPREM-UMR 5254), 371 Rue du Ruisseau, 40004 Mont de Marsan, France

**Keywords:** pistachio shells, organosolv, lignocellulose, nanofibers

## Abstract

Cellulose and lignocellulose nanofibrils were extracted from pistachio shells utilizing environmentally friendly pulping and totally chlorine-free bleaching. The extracted nanofibers were used to elaborate nanopaper, a continuous film made by gravimetric entanglement of the nanofibers and hot-pressed to enhance intramolecular bonding. The elaborated nanopapers were analyzed through their mechanical, optical, and surface properties to evaluate the influence of non-cellulosic macromolecules on the final properties of the nanopaper. Results have shown that the presence of lignin augmented the viscoelastic properties of the nanopapers by ≈25% compared with fully bleached nanopaper; moreover, the hydrophobicity of the lignocellulose nanopaper was achieved, as the surface free energy was diminished from 62.65 to 32.45 mNm^−1^ with an almost non-polar component and a water contact angle of 93.52°. On the other hand, the presence of lignin had an apparent visual effect on the color of the nanopapers, with a ΔE of 51.33 and a ΔL of −44.91, meaning a substantial darkening of the film. However, in terms of ultraviolet transmittance, the presence of lignin resulted in a practically nonexistent transmission in the UV spectra, with low transmittance in the visible wavelengths. In general, the presence of lignin resulted in the enhancement of selected properties which are desirable for packaging materials, which makes pistachio shell nano-lignocellulose an attractive option for this field.

## 1. Introduction

Since the beginning of time, humans have tailored new materials to resemble functionalities observed in nature. These improvements have taken into account the innovation and the amelioration of the wellbeing of humankind; however, the welfare of natural environments has become a secondary concern. With the arrival of the XX century and with the formation of the European Union, different movements evolved and gained the power to pressure governments to overcome such disinterest in the environment [1]. This had an essential role in the regulation of polluting materials and the diverse investments made to research and develop new materials from sources other than fossil resources, such as biomass.

The use of biomass conversion to obtain value-added products has been a significant research field in the materials industry because of not only the use of a naturally occurring composite (biomass) to elaborate new materials or to extract high-value chemicals but also because of the new field made accessible in matters of the management of soil, forests and agricultural waste [2]. A material that has been abandoned or poorly used, e.g., biomass to energy conversion via pyrolysis, has proven its usefulness for elaborating new materials with active functions [3]. Pistachio is a popular nut, originally from Western Asia; however, its salty and desert origin has made it adaptable to diverse environments throughout the world and now it is cultivated mainly in Iran, the Mediterranean Countries and the United States of America. In the case of Spain, the pistachio plantations have constantly been growing since 2017, according to the Ministry of Agriculture, Fisheries, and Food (MAPA). The exploitation of pistachio for food purposes leaves as byproduct shells, constituting around 45% of the nut, the peel and kernel representing the remaining 55% [4]. The high content of cellulose in pistachio shells (around 50% depending on the source) has made them an interesting feedstock for the elaboration of cellulose nanocrystals [5,6], pyrolysis-derived products [4], fermentable sugars [7] and essential oils [8], among others.

Among the different biomass materials, one of the most studied products has been nanocellulose. Nanocellulose appeared in 1983 as a fibrillar unit of cellulose isolated from cellulosic fibers through mechanical forces [9]. On its origin, this appeared to be an expensive process with low yields. However, since its first appearance, several factors have influenced the potential use of microfibrillated and nanofibrillated cellulose. The first is the boom of pulp in the paper and packaging industry, which has fueled the research in this field because of the reduction in basic pulp price, thus triggering high-value-added products to guarantee viability [10,11]. The second is the use of waste biomass as an attractive source of materials from renewable and local resources, as, with this underused cellulose, the prices would be lowered by broadening the lignocellulosic feedstock for such processes [12,13].

Moreover, as the fibrillation breaks the fiber down into its lower units, the traditional influence of the purity of the cellulose fibers and the need for strong and tough fibers is no longer the main objective when elaborating nanocellulose, as these fibers would be broken down, which has boosted further the search for new and interesting cellulosic sources. This, combined with different extraction methods, has given the nanocellulose world a vast and diverse range of shapes and properties [14]. Another contribution of nanocellulose to green chemistry relies on its inclusion within a multiproduct organosolv biorefinery since the nanocellulose manufacturing process offers the possibility to obtain different products based on the other macrocomponents of the lignocellulosic feedstock, lignin and hemicelluloses [15]. Traditionally, the organosolv pulp mill has been considered unviable because of the high installation cost and the resulting pulp properties compared with other industrially available pulps as sulfite and sulfate pulps. This apparent weakness has proven to be less remarkable at the nanofibrillated scale while also allowing defibrillation to occur easily, and the resulting nanofibers are of similar characteristics [16].

Nevertheless, the nanopaper world has traditionally conserved the desire inherited from the paper mills to obtain pure cellulose fibers of high whiteness and brightness [17]; moreover, the achievement of translucent and even transparent films has attracted interest in nanofibers as biobased building blocks for new composite materials [18]. However, in the last decade, researchers have explored the potential use of micro or nanofibrillated wood flour or partially delignified wood as a source of lignin-containing nanofibers, called lignocellulosic nanofibers (LCNF) [19]. The use of LCNF has been studied as a step before saccharification [20], as thermally performant materials [21] or as nanopapers with increased hydrophobicity [22].

In the present work, nanopapers from pistachio shells were elaborated for the first time. Moreover, the influence of pulping and bleaching on the final properties of lignocellulose nanofibers and the nanopapers elaborated from them in terms of morphology, physical and mechanical properties, and their appearance and potential applications, were evaluated.

## 2. Results and Discussion

### 2.1. Lignocellulose and Cellulose Nanofibers

#### 2.1.1. Chemical Composition of the Selected Biomass

**Figure 1:**
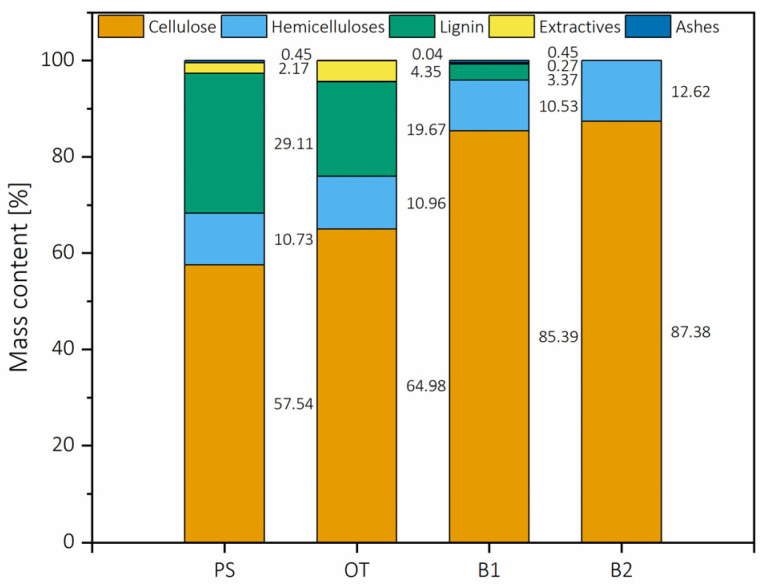
Composition of the pistachio shells through the different processes. PS is pistachio shells, OT is after organosolv treatment, B1 is after the first beaching, and B2 is after the second bleaching.

Nutshells have proven to be a rich source of cellulose in short size, which is a good feedstock to produce nanocellulose. The chemical composition diverges depending on the soil, the climate conditions and the inherent genealogy of the trees, having potentially more divergence than wood but similar to those of fibrous plants. In the case of pistachio shells, previous works have reported cellulose content to be 30–55%, hemicelluloses 20–32% and lignin 12–38% [5,8,23,24]. In the present work, cellulose was 57.54%, while hemicelluloses were 10.73%, and lignin content was 29.11%. The composition of pistachio shells throughout the processing is presented in Figure 1. Cellulose and lignin contents were similar to published works, while the hemicellulose content was lower than those mentioned above. Organosolv pulping resulted in a partial extraction of lignin, ≈33% of the total lignin content, with a more potent extraction during the first alkaline bleaching. For efficiency purposes, lignin was precipitated from organosolv black liquor and the black liquor of the first bleaching, totaling 24 g/100 g of the initial mass, representing a recovery of ≈82% of the total lignin content, thus resulting in an efficient, mild procedure to fractionate lignin from carbohydrates from pistachio shells.

Another remarkable aspect was that the process causes little harm to the hemicelluloses present in the pulp. If the ratio of cellulose/hemicelluloses present in PS were preserved in B2, the hemicellulose content would be 15.72%, while the hemicellulose content in bleached PS was 12.67%, thus representing a loss of ≈8% of the original hemicellulose mass.

**Figure 2:**
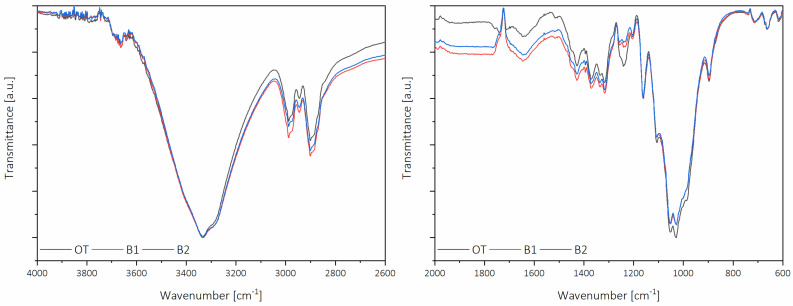
Infrared spectra of the different nanofibers.

Figure 2 presents the infrared spectra of pistachio shells after organosolv treatment and first and second bleaching. It can be appreciated that the spectra have few differences; however, differences are related to the presence of phenolic compounds identified as part of the lignin structure. The differences are mainly intensity in the 4000–2600 cm^−1^ range, particularly in the C-H stretching region in methyl and methylene groups. In the 2000–600 cm^−1^ region, called the fingerprint, more significant differences can be appreciated. In particular, there was a progressive loss in the band at 1240 cm^−1^, which corresponds to C-O stretching in the lignin aromatic ring, the band at 1110 cm^−1^, corresponding to the C-H aromatic deformations, and the band at 890 cm^−1^, corresponding to the aromatic C-H bending [15]. Considering the low amount of hemicelluloses solubilized during organosolv treatment and totally chlorine free (TCF) bleaching, there were no perceptible changes in the non-cellulosic polysaccharide regions.

#### 2.1.2. Morphology of the Elaborated Nanofibers

**Figure 3:**
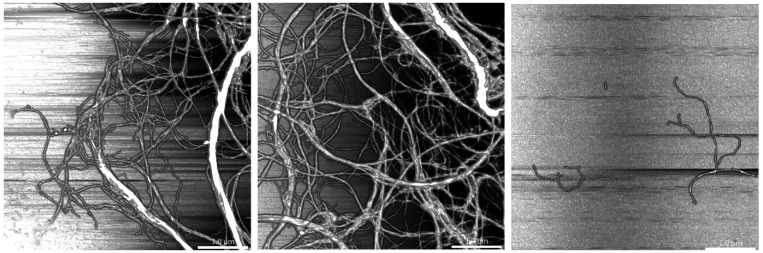
AFM images of the elaborated nanofibers. From left to right: OT, B1 and B2. Scale bar corresponds to 1 μm.

Figure 3 presents the AFM topographies of nanofibrillated samples. Nanofibers containing lignin presented higher resistance to defibrillation, which was visible in two phenomena, the first being the presence of larger fibers (poorly fibrillated) and the second being the entanglement of fibers. This highlights the notion that finding a cluster of nanofibers in B2 at the same concentration was harder than for OT and B1, with fibers being thinner, shorter and highly dispersed. The samples were processed under the same conditions to assess differences related to the raw material; thus, from the present images, it can be stated that B2 fibers would require fewer passes through the homogenizer to obtain similar results to those of OT and B1.

**Figure 4:**
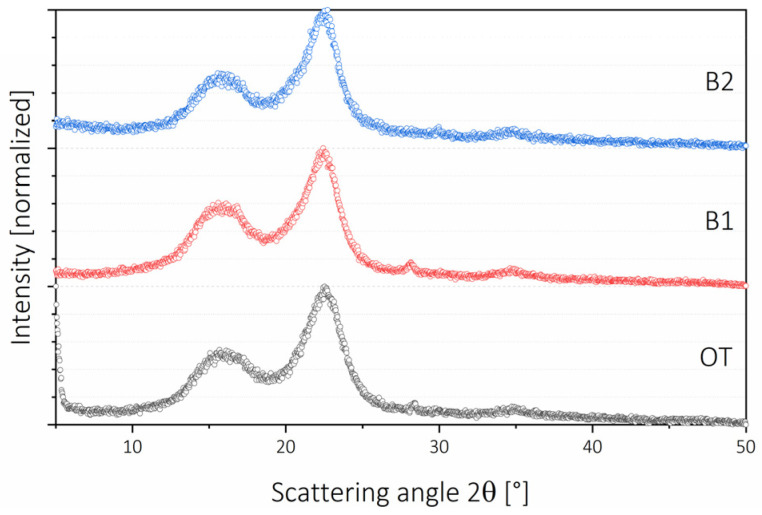
X-ray diffraction patterns of the elaborated nanofibers

Normalized XRD patterns are presented in Figure 4. There are low differences in terms of patterns. The most remarkable was the broader peak at the 200 planes for OT fibers (20% lignin) and the difference in the normalized prevalence of the same plane when comparing B1 and B2. The broadening of the OT signal is undoubtedly due to lignin, which is more evident in B1 and B2 samples, as both the overlapped 1–10 and 110 planes and the 200 plane present sharper peaks. This was related to the presence of a signal corresponding to lignin, which usually presents a broad and low diffraction pattern ranging from 10° to 35° [25]. In terms of crystallinity, it was calculated as 69.21% for OT, 82.20% for B1, and for B2, it was 85.38%. This was a high value compared with cellulose obtained from wood-based fibers but consistent with fibers from shells, which tend to have higher crystallinity indices [16].

### 2.2. Lignocellulose and Cellulose Nanopapers

#### Appearance and Color Properties

**Figure 5:**
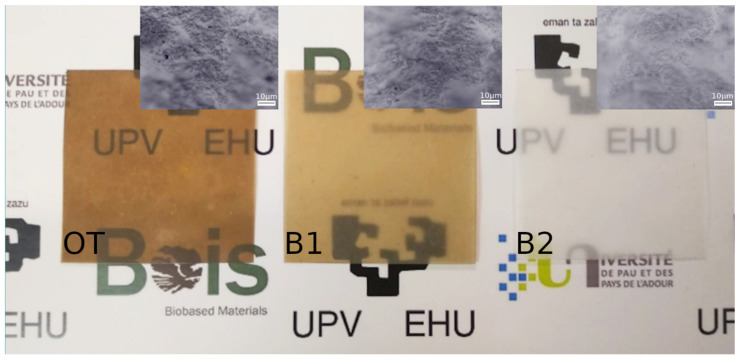
Visual aspect and micrographs of the elaborated films.

The visual aspect of the nanopapers is presented in Figure 5. While it is not perceptible at first glance, a second check comparing the “V” in samples OT and B2 gives significant information regarding the visual properties of these films. While the color of OT was darker due to the presence of lignin, the opacity of B2 was higher, even though the color was white. This has a first relationship with the thickness of the nanopapers, being 60 ± 0.2 μm for OT, 72 ± 0.1 μm for B1 and 74 ± 0.1 μm for B2. These differences are not to be neglected, but they occurred because of a major ductility during pressing, as the wet mats were formed with the same device, and the amount of dry matter was calculated for a nominal grammage of ≈95 g m^−2^. However, under the same conditions, samples containing lignin expanded more and formed nanopapers of 81, 78 and 97 g m^−2^ for OT, B1 and B2, respectively. This confirms the value of lignin as a plasticizing agent in biobased polymers, as stated before [26]. Moreover, the stronger interactions between B2 nanofibers resulted in thicker and less translucent nanopapers; these entanglements were perceptible from the micrographs provided in the insets.

**Figure 6:**
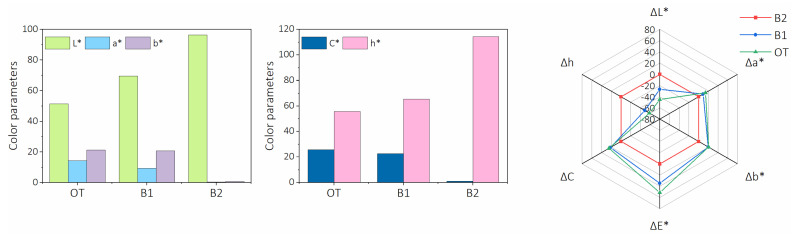
Color properties of the elaborated films.

Figure 6 presents the CIEL*a*b* color properties of the different nanopapers. The obtained lightness (L*) for the bleached nanofibers (96.45) was at the same level of fibers extracted with a similar method but from other raw materials [27], and it was higher than the minimum required by the paper industry (93), while for B1, it was 69.65, and for OT, it was 51.54. As can be seen, the coloring of nanopapers containing lignin did not meet the current requirements for lightness, a value related to the whiteness of the paper. Another value related to whiteness is the Chroma (C*), which indicates the intensity of the color and was reduced from 25.72 in the case of OT to 21.73 for B1 and 1.05 for B2. This indicated that, while there was a significant increase in the L dimension from OT to B1, the colors were still vivid, which can be related to the small change in the b* parameter (yellowness), which only descended from 21.32 in OT to 20.73 in B1.

**Figure 7:**
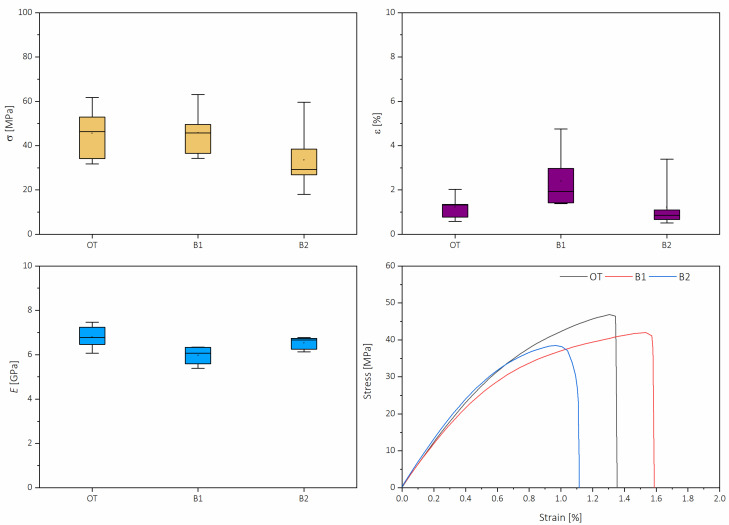
Modulus of elasticity (*E*), ultimate tensile strength (σ), strain at rupture (ε) and stress vs. strain diagrams (closest diagram to mean values).

Figure 7 presents the box plots for ultimate tensile strength (σ), strain at fracture (ε), Young’s modulus (*E*) and typical stress vs. strain diagrams of each set of papers tested. Box plots allow an understanding the maximum and minimum of each sample and the divergence of test specimens within each sample tested. In this plot, small squares indicate the mean values, the box represents the 25, 75 interquartile range, while the whiskers represent maximum and minimum. Ultimate tensile strength had a mean value of 45.54 MPa for OT, 45.82 for B1 and 42.65 for B2; on the other hand, strain at fracture was 2.37% for B1, 1.20% for OT and 1.13% for B2. It can be appreciated that, for σ, the ranges are more asymmetric. In contrast, for ε, the ranges are more homogeneous in the case of OT and B2, but also more symmetric in the case of B1, with the only remarkable aspect being the upper maximum, which was considerably higher, but this can be attributed to an isolated outlier. Tensile strength and strain at fracture are lower than values reported by other authors for lignocellulose nanopapers, in which these values range between 90 and 130 MPa for σ and between 1 and 6.5% for ε [28,29,30]. In the present work, the overall range of σ was between ≈17 and ≈63 MPa, while the ε was between 0.5 and 5%; in previous works, it has been stated that the preparation method might influence the mechanical properties of nanocellulose-based films [31,32].

In terms of elasticity, *E* had a mean value of 6.79 GPa for OT, followed by B2 with a mean of 6.53 GPa, and B1 with 5.97 GPa. Moduli had fewer divergences between samples, with median values close to the mean and the outliners close to the 25, 75 range, which means that the recorded values have more confidence than stress or strain alone. From the stress–strain diagram, it can be seen that differences were almost imperceptible within the elastic region at simple sight. When performing an ANOVA with the Tukey test, the significance between the moduli means at the 0.05 level was found only between OT and B1, while for the rest of the pairings, no significant differences were found. However, during the plastic deformation of the test, there were more notable differences: tensile toughness (*U*_T_), the energy absorbance of the nanopapers, while being subject to tensile stress, had the highest value for B1 (4.62 MJ m^−3^), followed by OT (4.09 MJ m^−3^) and B2 (28.91 MJ m^−3^).

**Figure 8:**
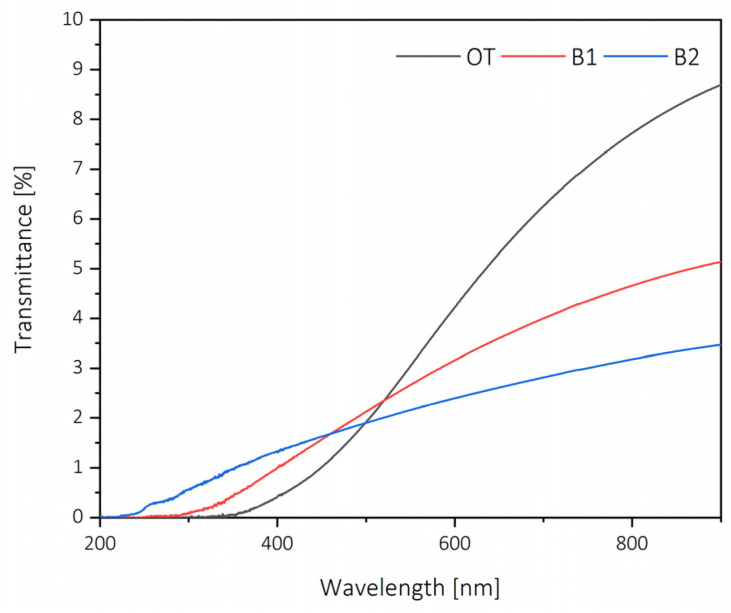
UV–vis spectra of the elaborated films.

Besides tensile strength, selected barrier properties were studied, notably UV resistance and surface free energy. UV–vis transmittance is shown in Figure 8; it can be appreciated that the nanopapers are rather more opaque than in other works [33]. This was mainly related to the manufacturing method; however, as it has been proven in other works [34], carbohydrate films containing lignin increase considerably the absorbance in the UV region (200–400 nm), with nanopapers containing 19% of lignin having an almost complete absorption in the UV range (%T at 400 nm of 0.33%). In the work by Gordobil and collaborators [30], in which they highlight the relevance of lignin as a UV-protecting agent, they found that the critical wavelength of emulsions having up to 2.5% of lignin increased considerably compared with the blank [35]. Another important value obtainable from the UV–vis spectra is transparency, which is defined as the logarithm of the transmittance at 600 nm, divided by the film thickness [36]. This value allows assessment of the property of the material regardless of its morphology, as is observed in Figure 5. For the elaborated films, transparency value was 8.67% for OT, 6.08% for B1 and 5.34% for B2, so once the thickness is taken into consideration, the visual transparency is corrected and it has a proportionally linear relationship with the lignin content. This opaqueness can be explained by the method selected for the production of nanopapers, as similar works tend to add less pressure and leave the hot pressing for longer times, or dry the film at room temperature prior to pressing [37,38]. However, the selected process intends to follow a potentially upscalable approach, consisting of different pressing cycles to dewater and then initiating the intramolecular bonding of lignocellulose fibers.

**Figure 9:**
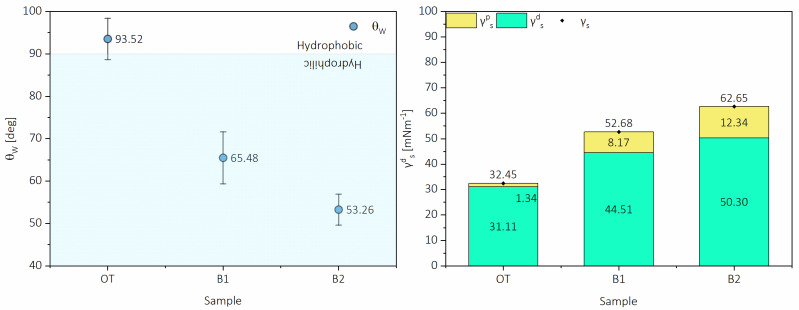
Water contact angle (θ) and surface free energy (γ) with polar and dispersive components.

The second barrier property tested corresponds to the surface wettability of the nanopapers. For this, contact angle measurements were performed, and the polar and dispersive components were calculated. These test results are presented in Figure 9, corresponding to the water contact angle and the surface free energy of each nanopaper. In the case of the water contact angle, it can be appreciated that the wettability of the papers is rather high. B2 nanopaper presented a water contact angle of 53.26°. At the same time, for nanopapers elaborated in the same way from different fibers, achieved values were 100.7° for blue agave bagasse obtained with an elemental chlorine-free bleaching process [39] and 93.7° with blue agave nanofibers obtained from a similar organosolv-TCF process [31]. However, in work by Sethi and collaborators [40], in which they studied nanopapers from an unbleached spruce pulp, the bleached nanopaper also had a low water contact angle. In contrast, the further addition of unbleached nanofibers resulted in a similar trend to increase surface hydrophobicity.

## 3. Materials and Methods

### 3.1. Obtaining Cellulose and Lignocellulose 

Pistachio shells were collected from commercially available sources, washed from salt and remaining peel and ground with a Retsch SM 100 mill with a 4 mm grid. As the first stage of the process, an organosolv treatment (OT) was followed to separate lignin from the rest of the shell. The selected conditions consisted of a 65:35 ethanol–water mixture and 0.05 M of MgSO_4_ as a catalyst, and the final liquid to solid ratio was 1:10. After organosolv processing, the total chlorine-free bleaching (TCF) was performed, starting with an oxygen alkaline bleaching stage (B1). For this, delignified shells were added to a NaOH solution (1:10 *w/v*) stabilized at pH ≈12; the reaction was performed at 98 °C for 60 min under an O_2_ atmosphere at 0.6 MPa, after which the sample was cooled to room temperature. The fibers were washed until neutral pH was achieved. As a final bleaching stage (B2), peroxide bleaching was performed with a 3 M solution of H_2_O_2_ stabilized at pH 11 with a blend of NaOH/Mg(OH)_2_ (3:1 ratio) and with 5 mmol of N,N-Dicarboxymethyl glutamic acid tetrasodium salt (GLDA) as a chelate; the reaction was done at 98 °C for 120 min under a 0.6 MPa O_2_ atmosphere. Organosolv treatment and TCF bleaching were done inside a 4 L Liter Zipperclave (Autoclave Engineers, Division of Snap-tite, Inc., Erie, PA USA) with PC-controlled stirring, pressure and temperature.

The different pulps were disintegrated with a high shear mechanical homogenizer (IKA T25 UltraTurrax, Staufen, Germany) at 15,000 rpm for 15 min, after which they were passed through a high-pressure homogenizer (GEA Niro Soavi PandaPLUS 2000, Parma, Italy) once with a pressure of 20 MPa, once with 50 MPa and, finally, ten times with a pressure of 60 MPa. Three different pistachio nanofibers were obtained, the first from organosolv treatment OT, the second after the first bleaching stage B1 and the third after the final bleaching stage B2. These were selected as being the most affected in their lignin content according to the process followed.

### 3.2. Elaboration of Lignocellulose Nanopaper

Nanopapers were elaborated to achieve a grammage between 70 and 80 g m^−2^. For this, a dispersion of lignocellulose nanofibers was vacuum-filtered with nylon membrane attached to a pore 3 Buchner funnel to form a wet mat. The wet mat was then hot-pressed under 110 °C following an increasing pressing cycle (1-2-3-5 MPa), with 1 min holding time at each pressure and a final curing pressure of 10 MPa for 5 min.

### 3.3. Characterization of the Obtained Nanofibers

The chemical composition of pistachio shells was analyzed throughout the whole process by following standard methods in terms of cellulose and hemicellulose content [41], lignin content [42], ashes [43] and solvent extractive contents with a modification of TAPPI T 204 substituting benzene with toluene [44]. Moreover, Fourier-transform infrared spectroscopy (FT-IR) was conducted with a Spectrum Two Spectrometer, equipped with a universal Attenuated Total Reflectance (ATR) accessory with an internal reflection diamond lens PerkinElmer (Waltham, MA, USA). The defined range of wavelength was from 4000 to 400 cm^−1^, the resolution 4 cm^−1^, and 30 scans were recorded for each sample.

The morphology and structure of the nanofibers were analyzed with atomic force microscopy and X-ray diffraction. AFM images were obtained operating in tapping mode with a scanning probe microscope, Nanoscope IIIa, Multimode™, Digital Instruments (Waltham, MA, USA), equipped with an integrated silicon tip cantilever with a resonance frequency of 300 kHz. X-ray diffraction was conducted with an X’Pert PRO multipurpose diffractometer, (Malvern Panalytical, Netherlands). Samples were mounted on a zero-background silicon wafer fixed in a generic sample holder, using monochromatic CuKα radiation (λ = 1.5418 Å) in a 2θ range from 5 to 50 with a step size of 0.026 and 80 s per step at room temperature.

### 3.4. Characterization of the Elaborated Nanopapers

The visual aspect of the nanopapers was assessed with a commercial camera; micrographs were recorded with an Eclipse E600 (Nikon, Tokyo, Japan) microscope working in reflection. CIEL*a*b* color properties were recorded with a PCE-CMS 7 ( PCE Instruments Albacete, Spain). The average values were used to obtain the differences between each color value (ΔL*, Δa* and Δb*), which corresponded to lightness, redness/greenness and yellowness/blueness.

Mechanical properties of the elaborated nanopapers were analyzed in terms of their tensile resistance. Tests were performed using a 5967 Universal Testing Machine (Instron, Norwood, MA, USA) equipment provided with pneumatic clamps and with a 250 N loading cell and a speed of 5 mm min^−1^. Samples were prepared, dog bone-shaped, 38 mm long, with a width of 5 mm and 0.065 mm thickness. The starting distance between the clamps was 20 mm.

Two barrier properties of the nanopapers were evaluated: UV resistance and surface wettability. The UV–vis light transmittance spectra were measured in a 200–900 nm wavelength range using a V-730 spectrophotometer (Jasco, Tokyo, Japan).The contact angle of the sessile drop was measured with an OCA20 contact angle system (Data Physics, San Jose, CA, USA). The Owens, Wendt, Rabel and Kaelble (OWRK) [45,46,47] method was used to determine the surface free energy with polar and dispersive components.

## 4. Conclusions

Nanopapers were prepared from three different pulps, consisting of partially delignified pistachio shells after organosolv treatment and after an alkaline oxygen bleaching sequence, and a totally delignified pulp after an hydrogen peroxide bleaching. The resulting nanopapers presented differences in their properties, with those containing lignin having better barrier properties against UV radiation and against water penetration (hydrophobicity), which are two important aspects for the packaging industry. In addition, mechanical properties revealed that a small amount of lignin results in tougher papers compared with bleached nanopapers, while an excess of lignin diminishes this benefit. This work showed the feasibility of using pistachio shells for producing nanopapers of competitive quality by giving added value to a subproduct; moreover, while the color changes are not negligible, the relevance of having fully transparent or white nanopapers is questioned in light of the benefits that small amounts of lignin have for the functional properties of these materials.

## Data Availability

The data presented in this study are available on request from the corresponding author.

## Sample Availability

Samples of the nanofibers and nanopapers are available from the authors.
